# Interactions of manganese with iron, zinc, and copper in neonatal C57BL/6J and parkin mice following developmental oral manganese exposure

**DOI:** 10.1016/j.dib.2017.10.050

**Published:** 2017-10-24

**Authors:** Melanie L. Foster, Thomas B. Bartnikas, Hailey C. Maresca-Fichter, Courtney Mercadante, Miriam Dash, Chelsea Miller, David C. Dorman

**Affiliations:** a North Carolina State University, College of Veterinary Medicine, 1060 William Moore Drive, Raleigh, NC 27607, USA; b Brown University, Department of Pathology and Laboratory Medicine, 70 Ship St., Rm. 522, Providence, RI 02912, USA

**Keywords:** Copper, Zinc, Iron, Manganese Toxicity, Mouse

## Abstract

High dose manganese (Mn) exposure can result in changes in tissue concentrations of other essential metals due to Mn-induced alterations in metal absorption and competition for metal transporters and regulatory proteins. We evaluated responses in mice with a Parkin gene defect (parkin mice) and a wildtype strain (C57BL/6J) following neonatal Mn exposure. Neonatal parkin and C57BL/6J littermates were randomly assigned to 0, 11, or 25 mg Mn/kg-day dose groups with oral exposures occurring from postnatal day (PND) 1 through PND 28. We report liver, femur, olfactory bulb, striatum, and frontal cortex iron, copper, and zinc concentrations and changes in hepatic gene expression of different metal transporters in PND 29 parkin and C57BL/6J mice. A companion manuscript (Foster et al., 2017) [1] describes the primary study findings. This data provides insights into strain differences in the way Mn interacts with other trace metals in mice.

**Specifications table**

**Table:** 

Subject area	Toxicology
More specific subject area	Toxicology of metals
Type of data	Figures and tables
How data was acquired	Spectroscopy (see materials and methods for instrument specifics)
Data format	Analyzed
Experimental factors	Tissue samples from neonatal mice given manganese
Experimental features	Liver, femur, olfactory bulb, striatum, and frontal cortex samples were analyzed to assess iron, zinc, and copper levels.
Data source location	Not applicable
Data accessibility	Data is within this article

**Value of the data**

• Developmental exposure to Mn in mice is associated with changes in liver iron concentrations.
• Changes in liver iron concentration are associated with changes in gene expression of *Hamp* in both wildtype (C57BL6) and parkin mice.
• Decreased hepatic expression of *Slc30a10* and *Slc40a1* were seen in PND 29 C57Bl/6J mice following neonatal exposure to 25 mg Mn/kg-day from PND 1–28. These changes were linearly correlated with liver iron concentration.
• While sample size in this study is small, the potential value of this data is that it includes an analysis of changes in trace element homeostasis following high dose Mn exposure in two mice strains, one of which has a defect in the Parkin gene.
• Our analysis may be useful as a reference for future, larger studies.

## 1 Data

We analyzed levels of iron, copper, and zinc in liver, femur, olfactory bulb, striatum, and frontal cortex in wildtype (C57BL/6J) and parkin mice after high dose oral Mn exposure during development (Table 1). Decreased liver iron concentrations were seen in PND 29 C57BL/6J mice given 25 mg Mn/kg-day when compared with controls. Decreased liver iron concentrations were seen in PND 29 parkin mice given either 11 or 25 mg Mn/kg-day when compared with controls. Liver copper concentrations seen in female parkin mice were higher (3.63 ± 0.21 μg Cu/g) when compared with male parkin mice (2.98 ± 0.11 μg Cu/g; p = 0.0099). Femur zinc concentrations seen in parkin mice were higher (83.7 ± 3.05 μg Zn/g) when compared with C57BL/6J mice (72.3 ± 2.10 μg Zn/g; p = 0.0030).

**Table 1:** Mean (± SEM) tissue zinc (Zn), copper (Cu), and iron (Fe) concentrations in PND 29 C57Bl6J and parkin mice following neonatal exposure to either 0, 11, or 25 mg Mn/kg-day from PND 1–28. Bold text indicates different from control (0 mg Mn/kg-day); italic text indicates control value in parkin mice different from wildtype (C57Bl6J). p ≤ 0.05.

	μg Zn/g			μg Cu/g			μg Fe/g		
	Manganese exposure (mg Mn/kg-day)			Manganese exposure (mg Mn/kg-day)			Manganese exposure (mg Mn/kg-day)		
**C57Bl6J**	0	11	25	0	11	25	0	11	25
Olfactory bulb	8.43 ± 1.07	9.09 ± 1.56	8.33 ± 1.60	2.84 ± 0.16	2.64 ± 0.17	3.04 ± 0.15	24.4 ± 1.46	23.5 ± 1.58	22.2 ± 1.92
Striatum	7.11 ± 1.36	6.01 ± 1.32^a,c^	9.33 ± 2.80	2.75 ± 0.10	2.79 ± 0.14	2.76 ± 0.09	16.0 ± 1.20	14.7 ± 1.17	13.7 ± 1.39
Frontal cortex	4.84 ± 1.15^b^	3.78 ± 0.50^d^	3.50 ± 1.83^c^	2.85 ± 0.19	2.92 ± 0.16	3.05 ± 0.22^a^	12.0 ± 1.21	11.5 ± 0.69^a^	11.0 ± 0.62^a^
Liver	24.5 ± 0.71	24.9 ± 2.20^a^	24.8 ± 1.30	2.97 ± 0.13	3.34 ± 0.21	2.93 ± 0.17	116.0 ± 11.6	91.6 ± 9.66	**56.1 ± 5.36**
Femur	74.5 ± 2.85	71.2 ± 3.48	71.4 ± 4.49	0.94 ± 0.07	0.94 ± 0.05	0.91 ± 0.04	29.0 ± 2.19	28.8 ± 2.88	24.8 ± 2.67
Sample size	10	10	11	10	10	11	10	10	11
**parkin**	0	11	25	0	11	25	0	11	25
Olfactory bulb	12.33 ± 5.30^a^	9.35 ± 1.68	7.65 ± 2.57	2.78 ± 0.26	2.82 ± 0.16^a^	3.07 ± 0.15	26.4 ± 2.07	22.3 ± 1.61	24.8 ± 2.37
Striatum	6.50 ± 1.75	4.09 ± 1.07^d^	9.34 ± 1.13^b^	2.66 ± 0.12^a^	2.97 ± 0.24^a^	2.71 ± 0.18^a^	13.1 ± 1.30	14.7 ± 1.50	10.6 ± 0.60
Frontal cortex	4.35 ± 1.36^a^	5.21 ± 2.98^e^	1.78 ± 1.15^b^	2.92 ± 0.30^a^	2.90 ± 0.26^a^	2.94 ± 0.20^a^	11.3 ± 0.81^a^	10.6 ± 0.75^a^	11.4 ± 0.71^a^
Liver	27.4 ± 1.78	24.8 ± 2.96	27.9 ± 1.71	3.29 ± 0.12^a^	3.11 ± 0.23	3.16 ± 0.19^a^	99.4 ± 12.6	**68.0 ± 6.24**	**59.9 ± 4.66**^a^
Femur	*80.9 ± 3.68*^a^	88.1 ± 5.01	81.8 ± 6.67	1.17 ± 0.10^a^	1.06 ± 0.08	0.97 ± 0.05^a^	32.7 ± 1.52^a^	33.7 ± 3.45	27.9 ± 2.84^a^
Sample size	10	10	10	10	10	10	10	10	10

a Single outlier removed from analysis

b *N* = 4 (insufficient tissue to complete analysis of all animals)

c *N* = 6 (insufficient tissue to complete analysis of all animals)

d *N* = 7 (insufficient tissue to complete analysis of all animals)

e *N* = 8 (insufficient tissue to complete analysis of all animals)

**Fig. 1:**
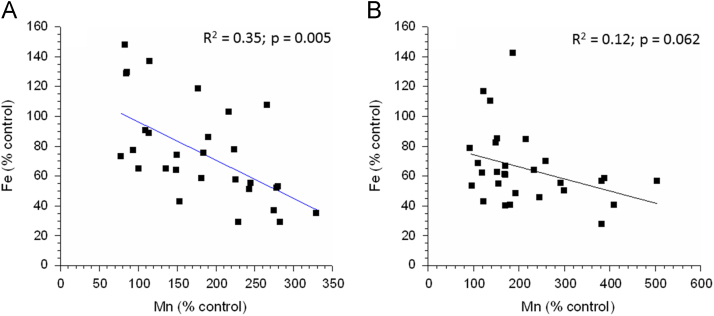
Change in liver iron concentration as a linear function of liver Mn concentration in wildtype (A) or parkin mice (B) following neonatal Mn exposure from PND 1–28.

Fig. 1 shows the inverse linear relationship between liver Mn and iron concentrations in the two mice strains. A statistically significant inverse relationship was seen in the C57Bl6 strain (*p* = 0.005) and a nearly significant relationship in the parkin mice (*p* = 0.062). An inverse relationship between hepatic Mn and iron concentrations has been seen previously [2], [3], [4], [5]. Mn and iron transport is regulated by transferrin receptor and divalent metal transporter-1 (DMT1), which is encoded by *Slc11a2* [6].

**Fig. 2:**
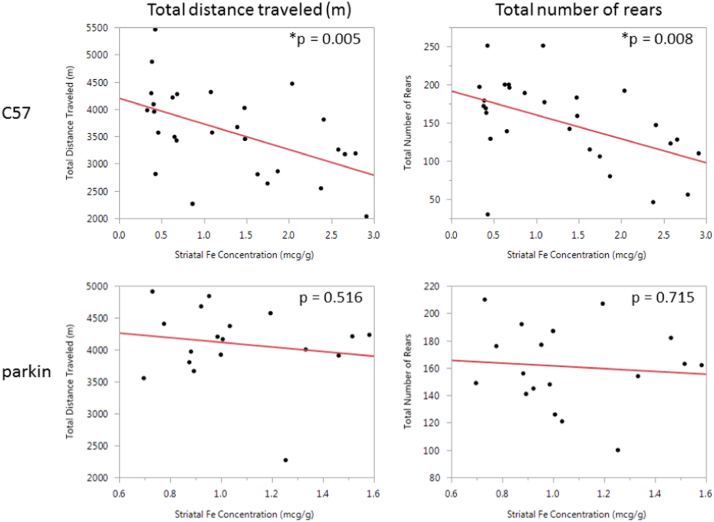
Change in spontaneous motor activity as a linear function of liver iron concentration in PND 29 wildtype (top) or parkin mice (bottom) following neonatal Mn exposure from PND 1–28.

Fig. 2 shows the inverse linear relationship between striatal iron concentration and motor activity in the two mice strains. A statistically significant inverse relationship for both measures of spontaneous motor activity (total distance traveled and number of rears) was only seen in the C57Bl6 strain (*p* = 0.005 to *p* = 0.008).

Significant linear correlations were seen between liver iron concentration and *Slc30a10*, *Slc40a1*, and *Hamp* expression in C57Bl/6J mice (Table 2). Significant linear correlations were also seen between liver iron concentration and *Slc40a1* and *Hamp* expression in parkin mice (Table 2). The protein product of *Hamp* (also known as hepcidin antimicrobial peptide), hepcidin, is involved in iron homeostasis, regulates iron storage in macrophages, and is critical for intestinal iron absorption.

**Table 2:** Mean (± SEM) liver gene expression (as % of control averaged across three measurements using different housekeeping genes) in PND 29 C57Bl/6J and Parkin mice following neonatal Mn exposure from PND 1–28 (see also [1] for more details). Bold text indicates different from control. *p* ≤ 0.05.

	Exposure dose (mg Mn/kg-day)			
**C57Bl6**	0	11	25	Correlation with liver Fe concentration
*Slc11a2*	100.0 ± 7.8	128.1 ± 9.3	99.4 ± 12.8	*p* = 0.846
*Slc30a10*	100.2 ± 12.5	75.2 ± 12.6	**42.3 ± 5.7**	**p = 0.004**
*Slc40a1*	100.0 ± 6.4	101.6 ± 8.4	**71.3 ± 11.8**	**p = 0.013**
*Hamp*	100.0 ± 11.1	124.6 ± 24.1	**25.7 ± 6.5**	**p = 0.001**
**Parkin**	0	11	25	Correlation with liver Fe concentration
*Slc11a2*	100.0 ± 4.2	75.0 ± 5.0	96.6 ± 14.2	*p* = 0.221
*Slc30a10*	100.0 ± 16.4	122.5 ± 10.4	85.7 ± 18.4	*p* = 0.256
*Slc40a1*	100.0 ± 10.1	82.5 ± 6.9	87.7 ± 12.5	*p* = 0.054
*Hamp*	100.0 ± 8.1	**43.5 ± 11.3**	**53.9 ± 16.6**	**p = 0.001**

**Fig. 3:**
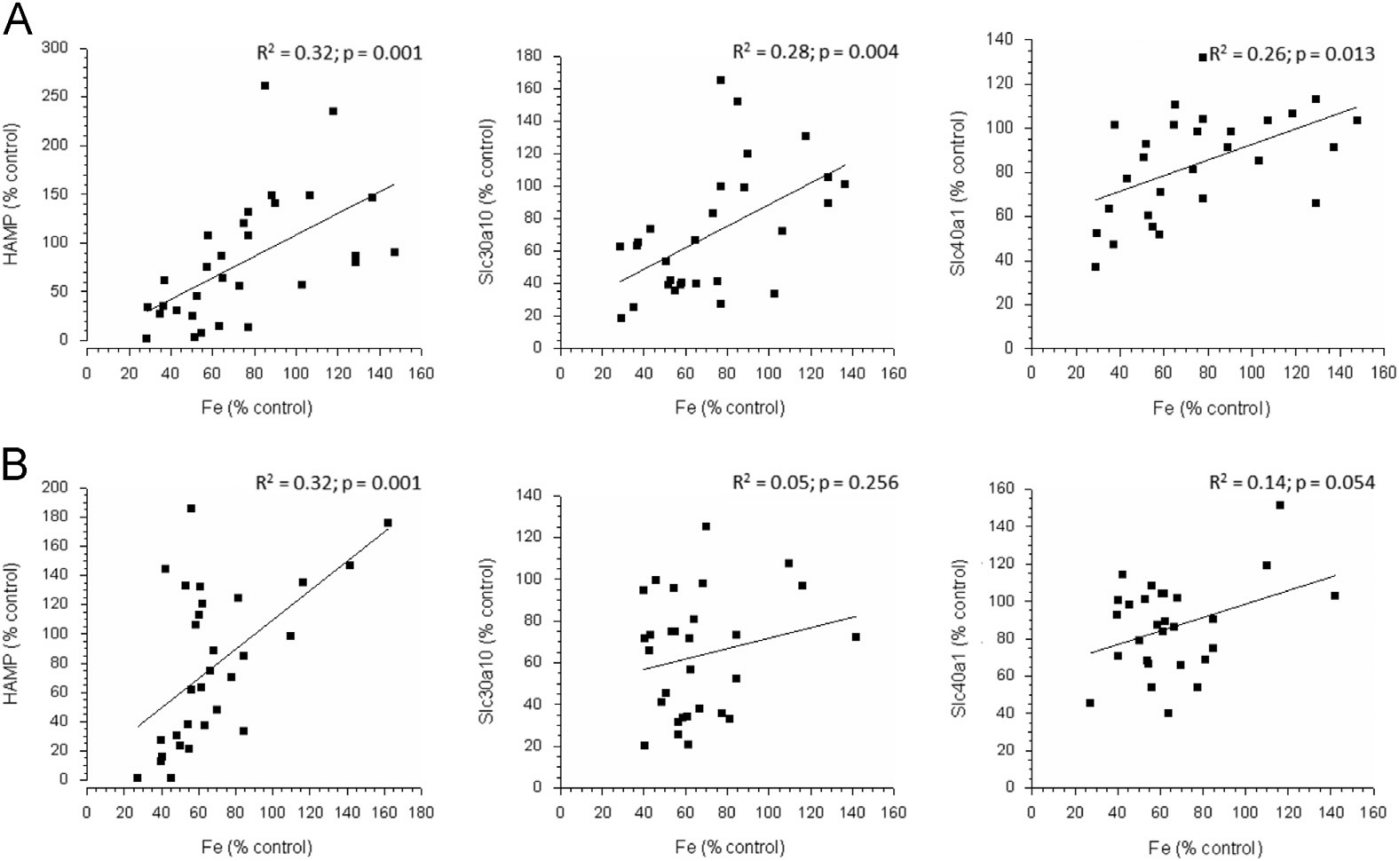
Changes in hepatic *HAMP* (left panel), *Slc30a10* (middle panel), and *Slc40a1* (right panel) gene expression (as % control) as a function of liver iron concentration (as % control).

Fig. 3 shows the linear relationship between liver iron concentration and *Hamp*, *Slc30a10*, and *Slc40a1* expression in the two mice strains.

**Fig. 4:**
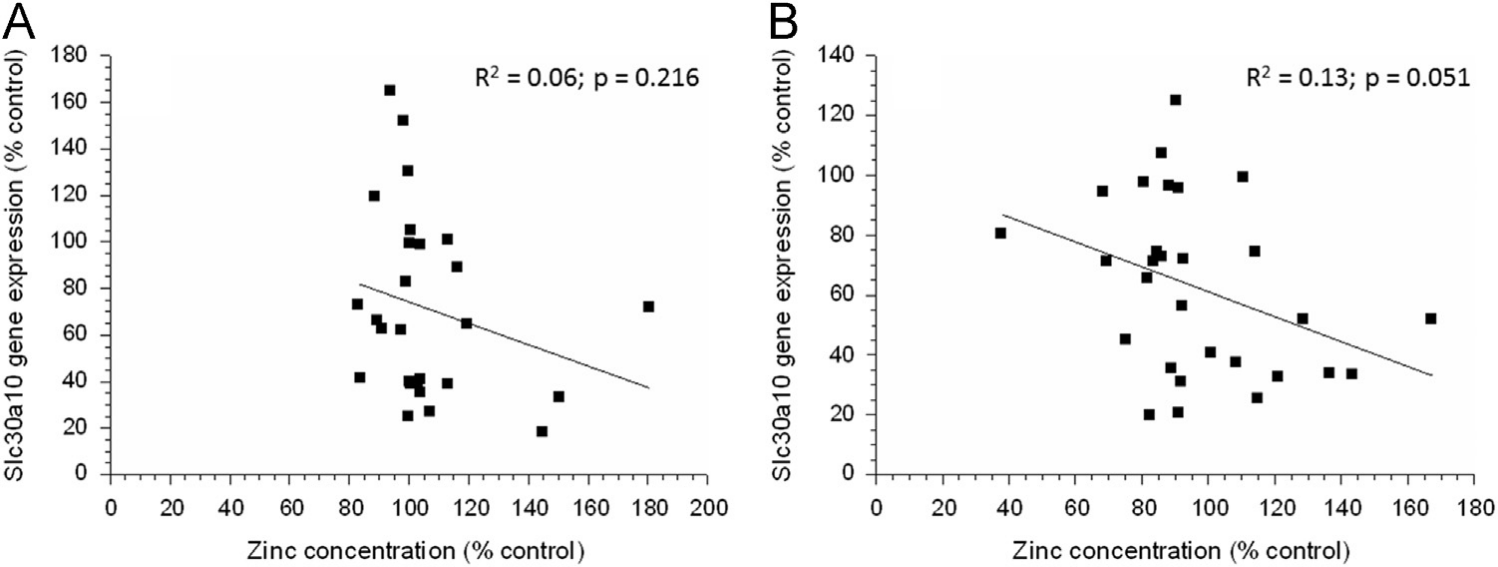
Changes in hepatic *Slc30a10* gene expression (as % control) as a function of liver zinc concentration (as % control) in C57Bl6 (A) and parkin mice (B).

Linear associations were not seen between liver copper or zinc concentration (as % control) and hepatic *Hamp*, *Slc11a2*, *Slc30a10*, *Hamp*, or *Slc40a1* gene expression (as % control) (data not shown). However, a near significant (*p* =0.0511) linear relationship was seen between liver zinc concentration and hepatic *Slc30a10* gene expression in the parkin mice (Fig. 4).

## 2 Experimental design, materials, and methods

### 2.1 Animals and animal husbandry

This study was conducted under federal guidelines for the care and use of laboratory animals (National Research Council, 2011) and approved by the North Carolina State University (NCSU) Institutional Animal Care and Use Committee. Mice were housed in the College of Veterinary Medicine's Laboratory Animal Resources (LAR) facility using standard caging systems and appropriate materials for enrichment. Animal rooms were maintained at daily temperatures of 22  ±  4 °C, relative humidity of 30–70%, and an air flow rate sufficient to provide 10–15 air changes per hour. Fluorescent lighting was controlled by automatic controls (lights on approximately 0700–1900).

Two strains of mice were used. C57Bl6 and parkin both were purchased from Jackson Laboratories (Bar Harbor, ME). The parkin mouse is an autosomal homozygous recessive knock out (Park2^t^^m1Shn^). On arrival, mice were individually housed in polycarbonate cages and provided food and water ad libitum. A pelleted, semi-purified AIN-93G certified diet from Bio-Serv (Frenchtown, New Jersey) formulated to contain 10  ppm Mn and 35  ppm iron was fed to all animals.

### 2.2 Breeding

Young adult (four-week-old) mice were purchased as breeding stock. A total of 10 female and five male mice of each strain was used. All mice were quarantined for at least one week prior to breeding. Harem breeding involving one male and up to two females was used. Each breeding harem was used up to three times. All females were assessed on a daily basis for the presence of a vaginal plug or abdominal enlargement consistent with pregnancy and on a weekly basis for weight gain. Pregnant animals were removed and individually housed prior to parturition. The day that pups were born was designated as PND 0. At parturition all mice were sexed and identified using paw tattoos. Experienced laboratory animal technicians that were not blinded to treatment groups performed daily cage side observations of all animals.

### 2.3 Neonatal Mn exposure

Littermates were randomly assigned to one of three dose groups (whenever possible one mouse/sex/litter/dose): control (0 mg Mn/kg-day), low (11 mg Mn/kg-day) and a high dose group (25 mg Mn/kg-day). Littermates were assigned to the highest Mn dose group (one mouse/sex/litter) then the next highest (one mouse/sex/litter) and finally to the control group (one mouse/sex/litter) until all pups were allocated. Litters and their dams were weighed daily, and the individual pup weight was used to calculate the Mn exposure dose. Aqueous Mn (MnCl_2_ • 4 H_2_O; Sigma Aldrich Chemical Corporation, St. Louis, Missouri) dosing solutions were prepared to provide 1 ml/kg dose volume to be given by mouth using a micropipette. All surviving mice were weighed on PND 28.

### 2.4 Motor activity

Mice (1 mouse/sex/litter/dose group) were evaluated for spontaneous horizontal and vertical movements on PND 29 prior to necropsy. For this test, individual mice were placed in a clean cage for 15 min during which their horizontal and vertical movements were monitored by infrared light beam sensors (Actimeter system; PanLab) and analyzed using Actitrack system software.

### 2.5 Necropsy and tissue collection

Necropsies were performed 20–24 h following the gavage exposure (or equivalent) after 28 exposure days. Mice were weighed and then euthanized using carbon dioxide inhalation and subsequent exsanguination. The following tissues were collected: striatum, olfactory bulb, liver, spleen, and femur for tissue iron, zinc, and copper determination. Samples of liver were also collected for gene expression studies. Tissue samples were transferred to plastic vials, frozen in liquid nitrogen, and stored at approximately −80 °C until subsequent analyses were completed.

### 2.6 Chemical analyses

Tissue samples were digested using a stepwise protocol: digestion in 0.2 mL 70% nitric acid and heating at 85 °C until dry, resuspension in 0.2 mL 30% Optima grade hydrogen peroxide (Fisher Scientific, Pittsburgh, PA) and heating at 85 °C until dry, repeated resuspension in hydrogen peroxide and heating until dry, resuspension in 0.2 mL 70% nitric acid and heating until dry, then a final resuspension in 1 mL 2% nitric acid. Samples were analyzed by graphite furnace atomic absorption spectrometry (GF-AAS) using an AAnalyst 600 spectrometer (Perkin Elmer, Waltham, MA) at the Environmental Chemistry Facility at Brown University using previously described methods [7]. In some analyses, the liver iron concentrations were converted to a % change from control (for each strain individually).

### 2.7 Quantitative polymerase chain reaction (qPCR)

To measure gene expression, all kits, reagents, and instruments were obtained from ThermoFisher and used according to the manufacturer's recommendations. RNA was isolated from thawed mouse tissues samples using Trizol then incubated with DNase I to remove genomic DNA. cDNA was synthesized from 1 µg RNA per sample using a High Capacity cDNA Reverse Transcription Kit. Gene expression levels were measured on cDNA aliquots using Taqman Gene Expression Master Mix and Gene Expression Assays on a Viia7 Real-Time PCR System using a relative standard curve approach. The following gene expression assays were used: Actb (Mm02619580), Gapdh (Mm99999915), Hamp (Mm04231240), Hprt (Mm03024075), Park2 (Mm01323528), Slc11a2 (Mm00435363), Slc30a10 (Mm01315481), Slc40a1 (Mm01254822). In some analyses, the gene expression data were converted to a % change from control (for each strain individually) and a grand mean calculated across the data sets normalized to different housekeeping genes (Gapdh, β-actin, and Hprt).

### 2.8 Statistical analysis

Individual data that appeared to be outliers were critically evaluated using a Dixon-type test for discordancy of an upper or lower outlier [8]. Statistical analyses were conducted using SAS Statistical Software (JMP, SAS Institutes, Inc., Cary, NC). Tissue metal concentrations, distance traveled, number of rears, and change in liver gene expression were initially analyzed using a mixed model with strain, sex, and dose as fixed effects and subject as the random effect. With few exceptions (e.g., liver copper concentration) a significant sex effect was not seen and data were pooled across sex in subsequent analyses. All data were subsequently analyzed individually by strain. Tissue iron, zinc, and copper concentrations, % change in liver gene expression for *Hamp*, *Park2*, *Slc11a2*, *Slc30a10*, and *Slc40a1* were inter-compared for the three Mn exposure groups by ANOVA or with a Welch ANOVA for unequal variances followed by a Tukey's honestly significant difference test to perform pairwise multiple comparisons. Correlation analyses between % change in either liver Mn or iron and % change in liver gene expression were calculated by linear regression of the plotted data points. A probability value of < 0.05 was used as the critical level of significance for all statistical tests. Unless otherwise noted, data presented are mean values ± standard error of the mean (SEM).
